# Reconceiving Reproduction: Removing “Rearing” From the Definition—and What This Means for ART

**DOI:** 10.1007/s11673-023-10281-4

**Published:** 2023-10-13

**Authors:** Georgina Antonia Hall

**Affiliations:** Faculty of Medicine, Dentistry and Health Sciences, The University of Melbourne, Children’s Bioethics Centre, The Royal Children’s Hospital, 50 Flemington Road, Parkville, Melbourne, VIC 3052 Australia

**Keywords:** Bioethics, Reproductive ethics, Reproductive rights, Medical ethics, Reproductive liberty, Assisted reproductive technology, Assisted reproductive treatment, IVF

## Abstract

The predominant position in the reproductive rights literature argues that access to assisted reproductive technologies (ART) forms part of an individual’s right to reproduce. On this reasoning, refusal of treatment by clinicians (via provision) violates a hopeful parent’s reproductive right and discriminates against the infertile. I reject these views and suggest they wrongly contort what reproductive freedom entitles individuals to do and demand of others. I suggest these views find their origin, at least in part, in the way we define “reproduction” itself. This paper critically analyses two widely accepted definitions of human reproduction and demonstrates that both are fundamentally flawed. While the process of reproduction includes the biological acts of begetting and bearing a child, I argue that it does not extend to include rearing. This reworked definition has little impact in the realm of sexual reproduction. However, it has significant ethical implications for the formulation and assignment of reproductive rights and responsibilities in the non-sexual realm in two important ways. First, a claim to access ART where one has an intention to rear a child (but does not beget or bear) cannot be grounded in reproductive rights. Second, lacking an intention to rear does not extinguish the reproductive rights and responsibilities for those who collaborate in the process. I conclude that clinicians collaborate in non-sexual reproduction at the point of triggering conception (begetting) and therefore have the right to refuse to be involved in non-sexual reproduction, in some instances, as do all reproductive collaborators.

## Introduction

The traditional ethical framing of reproduction defines the term as the “begetting, bearing and rearing” of children (O'Neill 1979, 25). This seminal definition was formulated by O’Neill in the 1970s, and tweaked by Robertson in the 1990s to become the “begetting, bearing or parenting” of a child (Robertson 1996, 39). This broadly accepted understanding is also the definition to which significant protections and entitlements of the reproductive right attach, at least in most Western liberal democracies. In this paper, I demonstrate that this definition of reproduction is flawed. I reject the inclusion of rearing (or parenting) within the reproductive paradigm, arguing that any “right to rear” claim sits outside its definitional scope. Rather, I suggest that rearing a child is the activity that follows on from the reproductive outcome—the birth of a child—but is not part of the reproductive process itself. The change occurs at the point that the baby is located outside the womb, when a qualitatively different process of rearing the baby begins. I propose a reformulated definition of reproduction as the begetting and bearing of a child. This means that reproductive rights only apply to individuals who beget and/or bear a child, thereby distinguishing the reproductive right from any “right to rear” or right “to found a family.” I additionally explore the moral relevance of removing rearing (or parenting) from the definition of “reproduction” by identifying and analysing two of the moral implications of doing so.

The moral implications of this small definitional tweak are profound and yet have been largely neglected in the literature to date. I suspect this is due to the lack of definitional clarity around the term itself. In this paper, I demonstrate that while a revised, stricter definition of reproduction has little bearing on the how reproductive freedom is understood in the sexual realm, it foreshadows fundamental changes to established moral theory regarding those who hold reproductive rights and bear reproductive responsibilities in non-sexual reproduction. In particular, I suggest it has two important and morally relevant implications in this realm: 1) those with an “intention to rear,” but who do not beget or bear a child, do not “reproduce,” and 2) those with “no intention to rear,” but who collaborate in begetting or bearing a child bear rights and responsibilities in the reproductive process. In other words, reproductive rights and responsibilities are generated by collaboration in begetting and bearing a child, not by one’s intention, or lack of intention, to rear the resulting child.^1^

I draw two conclusions that have implications for clinical decision-making regarding provision of treatment; first, those seeking ART who do not beget or bear the child cannot ground their claim to access ART in the significant protections of the reproductive right. In other words, they cannot claim access to ART as part of their reproductive right. Second, clinicians bear a measure of moral responsibility for the child born of ART. This is due to their collaborative involvement in begetting a child and puts them on an equal moral footing to anybody else asked to join a reproductive process at the point of triggering conception. These conclusions support the concluding claim of this paper that clinicians are morally entitled to agree, refuse, or place conditions on their involvement in ART. In other words, it is ethically justifiable for clinicians, in some instances, to refuse ART without violating a hopeful parent’s reproductive right.

## What is Reproduction?

Reproduction is a basic biological function which results in the birth of offspring. Defined as the biological process whereby “new individuals” are created by “parent organisms,” the process can occur sexually or asexually (Martin and Hine 2015). In this paper I confine my analysis to human reproduction and not that of other species. In human reproduction, every child born has two biological parents—the gamete contributions of a male sperm and female ovum. This biological process requires fusion of the male and female gametes, implantation of the zygote into a female uterus, and gestation of the fetus within the female uterus. This leads to childbirth, which produces live offspring organisms whose genetic characteristics are derivative of those parent organisms. Human reproduction is thus a physiological process which consists of a series of necessary and interconnected steps, a specific chain of physical events which need to be successfully achieved in consecutive order to create a child: fertilization, implantation, gestation, and childbirth. Since the late twentieth century, human reproduction occurs via two different methods. Reproduction that occurs as the result of sexual intercourse between a person with biologically male sexual characteristics, specifically, viable sperm, and a person with biologically female sex characteristics, specifically, viable eggs and a functioning uterus, is referred to in this paper as “sexual reproduction.” Reproduction that occurs with technical medical intervention (without sexual intercourse or privately administered artificial insemination) is referred to as “non-sexual reproduction”.

Medicalized non-sexual reproduction, known as ART, developed in the latter half of the twentieth century and inexorably altered the reproductive landscape. It is defined as

… procedures that include the in vitro handling of both human oocytes and sperm or of embryos for the purpose of establishing a pregnancy. This includes, … in vitro fertilization and embryo transfer, gamete intrafallopian transfer, zygote intrafallopian transfer, tubal embryo transfer, gamete and embryo cryopreservation, oocyte and embryo donation, and gestational surrogacy. (Zegers-Hochschild, Adamson, and de Mouzon 2009, 1520)

ART creates alternative reproductive pathways which circumvent or sidestep infertility, utilizing medical intervention to trigger non-sexual conception. The process requires the contribution of male and female gametes, implanted into the person who gestates and bears the child, a biological female with functioning uterus (hereafter referred to as “female”). Non-sexual reproduction is another pathway to parenthood for those seeking to rear a child. It is possible for people to have a genetic or gestational connection with the offspring by using their own gametes or (for a female) gestating and bearing the child. In this paper I will use the term “hopeful parents” to refer to those seeking to rear a child created by either sexual or non-sexual reproduction, while recognizing that this term could equally apply to those seeking to rear a child or found a family via other methods that do not involve a genetic or gestational connection with the child to be reared, such as adoption or foster care. I will explore the moral position of those who collaborate in the creation of a child at the point of triggering conception non-sexually. I will not consider the moral position or connection between those who beget or bear a child with no intention to rear the resulting child as the scope of this paper is limited to determining who holds reproductive rights and bears reproductive responsibilities, not exploring the grounds upon which rearing (or parenting) rights are assigned.

## The Ethical Framing of “Reproduction” in the Literature

In 1979 O’Neill described reproduction in a moral context as the “begetting, bearing and rearing” of children (O’Neill 1979, 25). “Begetting” describes the trigger of the process and first two biological steps on the sexual reproductive continuum: sexual intercourse between a male and female, followed by fertilization and implantation, where a sperm successfully fertilizes an egg in utero and implants in the female’s uterus. “Bearing” describes the next two steps in the process: gestation and childbirth, undertaken by the female partner of the sexual union. The final step of reproduction is defined as “rearing,” wherein the child born is raised by its genetic and gestational parents, who are typically recognized as the child’s legal guardians. In O’Neill’s account, all three components—begetting, bearing, and rearing—form part of the reproductive process. This definition of human reproduction was broadly accepted because at this time rearing rights and responsibilities were normatively assigned to those who collaborated in the biological process of sexual reproduction which was the standard—indeed the sole—method for humans to reproduce, as had been the case for millennia. (Aside from non-medical artificial insemination, which I specifically do not discuss here.) Therefore, the steps of begetting and bearing, followed by rearing one’s own genetic and gestationally related children, was the normal sequence of events, and therefore rearing came to be included as part of reproduction, without critical analysis. I reject O’Neill’s inclusion of rearing within the definitional frame of reproduction and demonstrate in this paper that this definition erroneously conflates creating a child with raising one. It also ignores the myriad situations wherein grandparents, older siblings, nannies, or non-biological parents might rear a child not biologically related to them.

By 1996 the term “reproduction” had evolved in the literature from O’Neill’s original definition. With the IVF industry offering new pathways to parenthood, Robertson reworked O’Neill’s definition, making one seemingly small amendment which, in theoretical terms, has a profound effect. Robertson redefines reproduction as the “begetting, bearing *or* parenting” (my italics) of children (Robertson 1996, 39). I suggest this tweak is a response to the fundamental changes that take place when reproduction occurs non-sexually. In this method, more people are involved at various points in the reproductive process, such as clinicians intervening at the begetting stage and the use of donor gametes and/or a surrogate. By simply replacing the “and” for “or,” Robertson positions each of O’Neill’s three interlinked components of reproduction as independently eligible for inclusion within the definitional scope of the term “reproduction” when on their own, as when combined. In so doing, Robertson establishes each of the three actions as “reproductive” acts, whether performed in concert (as occurs in sexual reproduction) or separately (as in non-sexual reproduction). This reformulated definition recognizes and accounts for the fragmentation of human input that occurs in the non-sexual reproductive realm at various steps. It recognizes that those who beget or bear may not be the same as those who end up rearing the child. It recognizes the reproductive intent of those who, say, do not contribute a gamete to the process, or of a female who does not gestate the child she intends to rear. It also recognizes instances where an individual or couple intend to rear a child whom they neither beget nor bear, in situations such as using a donated embryo and surrogate.

This definitional reformulation may appear on the surface to be a simple tweak of O’Neill’s wording; however, I suggest the implications for moral theory are substantive. In his small change to O’Neill’s wording, Robertson holds that to beget, bear, *or* rear a child is “to reproduce.” In other words, regardless of whether or not a hopeful parent is involved in either or both of the preceding steps of begetting and bearing, their “rearing” ought morally to still be eligible to qualify for recognition as “reproduction” or a “reproductive” act. Robertson argues that individuals can seek to ground a claim to rear a child in their “procreative” (or reproductive) liberty, stating that, “Claims of procreative freedom logically extend to every aspect of reproduction: conception, gestation and labor and childrearing” (Robertson 1983, 408). This argument has the effect of assigning the substantive and broadly recognized moral protections of “reproductive liberty” to the pursuit of “rearing” a child, regardless of whether or not this step is preceded by the begetting or bearing stages as occurs in sexual reproduction. I reject Robertson’s claim that childrearing is a logical extension of procreative freedom.

## How “Begetting, Bearing and Rearing” Frame Reproductive Rights and Duties in the Literature

Robertson argues that reproduction is “fundamentally important to human flourishing” (Robertson 1983, 408) because of the many personal and societal “goods” the process and its outcome provides. Within this list of goods, he includes “the genetic, biological, and social experiences that comprise it” (Robertson 1983, 408). This demonstrates conflation in the discourse of the reproductive stages of begetting and bearing children with the rearing of them. On this view, the recognition and universal respect for reproduction is largely grounded in the importance of parenthood as a meaningful, valuable, and significant human experience (Robertson 1996). Robertson’s view is broadly accepted in the literature. Harris, for example, similarly argues that “having children” is more morally significant than the exercise of a bare preference (Harris 2007). He defines reproduction not only as the biological process of creating a child but of “having children,” rearing them, again framing rearing as sitting within the reproductive paradigm, noting that “the right or freedom to found a family expresses something so basic and deep-rooted in human psychology and social practice that it seems hardly worthy of special attention” (Harris and Scarre 1989, 133).

The idea that people should be at liberty to make private and autonomous decisions to “found a family,” is a central concept of international human rights dialogue (United Nations General Assembly 1995, 40; United Nations General Assembly 1948, art.16.1). The law in most jurisdictions also recognizes and enshrines protections of reproductive freedom, which extends to include rearing within its definitional frame, as noted by Montgomery in 1970: “That there is a ‘right’ to found a family and have children cannot be seriously questioned” (Montgomery 1970). Again, the focus of the attention is on the outcome of the process, that is, the creation of a family rather than the biological stages that bring this about. Early Western legal interpretation of reproductive autonomy and the legal definition of reproductive interests was primarily formed in the United States in the context of *avoiding* having children.^2^ The legislature has been slower enshrining and protecting the rights of individuals *to* reproduce. Interestingly, the majority of legal cases in the United States examining the right to reproduce focus on the “rearing” aspect. Furthermore, within that narrow focus, only one aspect of rearing has been addressed by the judiciary—the right to parent in one’s own way—not the right to *become* a parent.^3^

In response to the habitual conflation in the literature of the reproductive right and the right “to found a family.” I seek to distinguish between these two rights and suggest they are not interchangeable as framed in the literature. The right to “found a family” is enshrined in the human rights discourse, notably in Article 16 of the United Nations Universal Declaration of Human Rights, which states, “Men and women of full age, without any limitation due to race, nationality or religion, have the right to marry and to found a family.” This wording does not distinguish between a right to reproduce and a right to rear. In this paper I take the right to reproduce to be the individual freedom to use one’s body and body parts to try to reproduce. I have argued in an earlier paper that the reproductive right is no more than the right to try to reproduce, as reproduction as a biological process is collaborative, and therefore no individual is able to reproduce on their own (Hall 2022). They have the right to beget a child, that is, to have sex and to use one’s gametes for reproductive purposes (either to experience reproduction themselves, including, for some, the desire to parent a child or to contribute to the reproductive process or parenting project of others) and/or to bear a child, that is, the right to gestate and give birth to a child (to parent oneself, or for others as is the case in surrogacy).

The dictionary definition of “to found” (a family) means “to bring into existence” (Cambridge advanced learner's dictionary 2003). I take the right “to found a family” to mean the right to create a family or bring a family into existence. It is akin to a “right to rear” because forming a family is something that is not confined to being the result of reproduction, as noted earlier in cases of adopting or fostering a child. Any child born will need to be raised, but that does not mean that reproduction and rearing are the same thing. For example, one may “reproduce” non-sexually via gamete donation or surrogacy with no accompanying intention or desire to “found a family.” Therefore, while the *intent* of participating in the reproductive process is often “to found a family,” one could equally reproduce non-sexually with no such intent. The two are not the same. For that reason, any rights protections attached to either reproduction or founding a family are separate and distinct. Therefore, while I accept that, historically, the right to reproduce has been inextricably intertwined with rearing a child and forming or building families, they are distinct rights and the claim of individuals to reproduce (as opposed to a claim to rear a child or found a family) is grounded in a separate moral foundation. In this paper I will not discuss the form and strength of the claim to rear a child or to found a family, while acknowledging that such claims exist.

With reproductive freedom framed within Harris’s paradigm of a “basic human right” (Harris 2007, 79), the predominant position in the literature supports Robertson’s call for the presumptive priority of reproductive liberty against competing claims. Robertson holds that reproductive liberty should be protected and promoted in all instances unless there is compelling contrary evidence, for example, evidence-based harms to the future child. Harris builds upon this view, adding that the “democratic presumption” in favour of personal liberty places the burden of proof in the hands of those seeking to constrain reproductive freedom (Harris 1998). He argues that limits to procreative liberty “must amount to high probability of major harm to potential children” (Harris 2004, 75) to override the fundamental importance of reproductive pursuit. Both Robertson and Harris position the reproductive right as virtually absolute, arguing that there are few situations of harm to either a gamete donor, surrogate, or to the future child born of treatment where such liberty could justifiably be overridden (Robertson 1996, 4, 122; Harris 2007, 74).

In the non-sexual realm, scholars argue that the broad scope of freedoms offered to those who reproduce sexually carry across into the non-sexual realm as the freedom to access ART. They argue that such unfettered access forms a part of reproductive freedom itself. For example, Robertson reasons, “if bearing, begetting or parenting children is protected as part of personal privacy or liberty, those experiences should be protected whether they are achieved coitally or noncoitally” (Robertson 1996, 4). The moral rationale is that since all individuals are equally capable of feeling a keen reproductive *desire*, that any reproductive method utilized to pursue this goal is morally equivalent. In other words, the reproductive *method* utilized is morally irrelevant. Harris argues that access to ART ought similarly to be defended on the grounds of the presumptive primacy of reproduction (Harris 2007, 74). Robertson positions accessing ART as something that sits within the realm of a hopeful parent’s decisional autonomy: “… individuals should be free to use these techniques or not as they choose … unless strong justification for limiting them can be established” (Robertson 1996, 4).

Jackson strongly rejects any restrictions to ART access, arguing that it is manifestly “unfair to take advantage of the opportunity afforded by their biological incapacity in order to assess the wisdom of an infertile couple’s decision to start a family” (Jackson 2002, 178). Jackson’s focus on “starting a family” emphasizes the rearing aspect of reproduction and seeks reproductive protections for parental freedom. While there may be a right to found a family or a right to rear, it is distinct from the reproductive right if one does not beget or bear the child. If rearing sits outside of the definition of reproduction, then having an intention to rear, but not participating in either the begetting or bearing stages of a reproductive process similarly sits outside of the reproductive rights paradigm.

This theoretical framing leads to the predominant position in the literature that denial of ART is discriminatory and violates the reproductive right.^4^ Implicit in the argument that ART access ought to be at the discretion of hopeful parents is the assumption that the clinician has no moral role in the process. For example, Robertson argues that reproductive liberty “should be equally honoured when reproduction requires technological assistance” (Robertson 1996, 4), which frames the role of the clinician as a mere technical operator. Such a view defends an extension of the scope of reproductive liberty of hopeful parents to include ART access on the grounds they “deserve the presumptive respect that decisions about coital reproduction garner” (Robertson 1995, 1024). I argue that this position conflates the negative rights assertion of reproductive freedom with a positive right to access services, and I suggest that this stems from the conflation of reproduction with “founding a family.” The two need to be separated and recognized as distinct. I will now formulate a revised definition of reproduction that takes account of this distinction.

## Removing “Rearing” From the Definition of Reproduction

I argue against both O’Neill and Robertson’s definitions of reproduction on the grounds that they go beyond the scope of the reproductive process itself. I suggest that rearing does not form a part of the reproductive process because the point at which the reproductive process concludes is the point at which the child is born. Reproduction includes the begetting and bearing of a child; begetting is the physical act that triggers the biological stages of fertilization and implantation. It is the act of sexual intercourse in sexual reproduction, and the provision of ART treatment in non-sexual reproduction. Bearing concerns the gestation within a female’s body and childbirth. The birth of the child is the completion of the process; it is the *outcome* of the reproductive process. While rearing may be the desired and intended outcome that follows on from the process, I argue that it does not form part of reproduction itself. In short, I argue that reproduction is a distinct set of interconnected biological steps, with the outcome of the birth of a child. Rearing is a separate set of actions that may, indeed, *follow on* from the outcome of reproduction but do not form part of reproduction *itself*. Rearing is a separate moral paradigm.

I propose a refinement of O’Neill and Robertson’s definitions of reproduction, removing rearing from within the definitional scope:

Reproduction is the begetting and bearing of a child who is born.

I suggested earlier in this paper that O’Neill’s inclusion of “rearing” in her definition is understandable because the period in which she formulated the definition (O’Neill 1979) was prior to the widespread use of ART. In the sexual realm, rearing rights in most Western liberal nations are presumptively awarded to those who beget and bear the child. Indeed, it makes moral sense for the conferral of parental entitlements and obligations to be awarded to those with biological genetic and gestational connection to the child. The genetic and gestational roles are always and only shared between a male and female of a sexual union. Regardless of whether the male or female intend to become the parents of a child that is born to them, they are biologically, socially, and legally regarded as the child’s parents upon birth, due to their collaboration in the sexual reproductive process. (In this paper I limit consideration to situations wherein sexual intercourse is consensual and the reproductive outcome is the shared desire of both parties, while accepting that this is not always the case where there are situations of trickery or rape or where one partner is under the age of consent.) In order to divest themselves of unwanted parental rights and responsibilities, they must formally and legally sign over these rights to the state or another legal guardian. Reproduction and rearing are therefore inextricably bound in sexual reproduction, but I have demonstrated they are not the same thing.

Indeed, the acts of begetting and bearing children are not the sole ways to become parents. Consider adoption or foster care, which confer rearing rights and responsibilities upon individuals or couples who have not been involved in begetting or bearing the child they raise. One cannot ground a claim to adopt a child in one’s reproductive rights. It could be grounded, perhaps, in a strong desire to parent a child or to found a family. While there may be compelling moral reasons to recognize, protect, and promote such interests, it is nonetheless distinct from any reproductive right. It is clear that adoption is not a form of reproduction*.* Adoption connects the desire (of a hopeful parent) to rear a child with the practical *outcome* of childrearing. While these two actions may recognize and operationalize the broadly experienced desire to rear a child, that process does not include the steps of genetic or gestational connection with the child who is reared—that is—the reproductive steps. Adoption demonstrates that there is good reason to distinguish between reproducing and rearing a child, quite aside from considering the issue in relation to ART.

## The Ethical and Practical Implications of a Stricter Definition of “Reproduction”

Defining “reproduction” is an interesting exercise in the sexual realm, but as argued above, it has negligible practical impact on the way reproductive rights and rearing rights are understood and accepted for a child produced sexually. This is because the right to rear is historically borne out of the reproductive process. Whether these two rights are separate or combined is immaterial in this realm as they are inextricably intertwined—one always normatively follows the other. However, I argue that the conflation of the two rights into one is a conceptual mistake inherent in our framing of reproductive rights in moral theory. This conceptual error becomes clearer and has substantial ethical and practical implications in the non-sexual realm—in particular, for the grounds upon which a hopeful parent can access ART and for the assignment of rights and responsibilities for all who collaborate in begetting and bearing a child via ART. I suggest theoretical confusion exists over the scope of actions and the entitlements that reproductive rights cover in the non-sexual realm, which has led to flawed arguments regarding the moral basis for ART access and the obligation of clinicians to assist the process.

I argue there are two key ethical implications of reframing the reproductive paradigm as the begetting and bearing of children, and they have practical implications for ART provision. First, removing “rearing” from the reproductive paradigm distinguishes the right to reproduce from the “right to rear” or the right “to found a family.” This matters ethically because it means that those who have an intention to rear a child via ART but who do not beget or bear (i.e. have genetic or gestational input into the process) cannot be said to have reproduced. The practical implication of this ethical argument is that those who do not collaborate in begetting or bearing a child non-sexually cannot ground a claim to ART access in their reproductive rights. An intention to rear does not generate reproductive rights. Second, the lack of an intention to rear a child does not extinguish rights and responsibilities toward the child who is created. This is ethically important because it means that those who collaborate in begetting or bearing a child non-sexually could have moral duties towards those implicated in and impacted by the provision of ART, including the child who is born of treatment. Broadly speaking, these responsibilities can be fulfilled by passing them onto another adult who is willing and able to rear the child (Fuscaldo 2006). In the non-sexual realm, this includes clinicians who provide ART treatment. I will now discuss each of these ethical arguments in turn and analyse the practical implications for ART.

## The “right to rear” is distinct from the reproductive right and having an intention to rear does not generate reproductive rights.

Robertson argues that any individual can claim to have reproduced without having participated in all three reproductive steps of begetting, bearing, or rearing a child, noting “it is meaningful to say that one has reproduced when one has merely passed on genes and neither gestated nor reared the resulting child” (Robertson 1983, 409). I accept this is a reasonable position to take on begetting a child. However, I reject Robertson’s extension of this argument which holds that reproductive protections ought to extend to rearing within that paradigm:

By the same token, we recognise parenting as an essential aspect of reproduction. Childrearing is a rewarding and fulfilling experience, deserving respect whether or not the person who rears also provided the genes or bore the child. To deny someone who is capable of parenting the opportunity to rear a child is to deny him an experience that may be central to his personal identity and his concept of a meaningful life.” (Robertson 1983, 410)

In framing childrearing as part of reproduction, Robertson assigns to parenthood the substantial protections afforded to the “presumptive primacy” of reproduction.

I suggest that this is dangerous ground. I do not dismiss rearing as unimportant; indeed, I recognize it as an immensely fulfilling and valuable lifelong human project. However, my point is that regardless of whether rearing is highly valuable, or indeed has intrinsic value, *it is not reproduction*, and therefore is unable to be accorded reproductive protection in the same way that the precursor steps of begetting and bearing a child can be.

I am arguing that a hopeful parent cannot ground claim to ART access in reproductive rights for those with solely an intent to rear. This is due to biological fact. For example, consider a single, infertile male who seeks to become a parent. He has a right to found a family; however, he is both physically and socially unable to make this happen via sexual reproduction. The options available to him in the non-sexual realm would be to acquire male and female donated gametes and to have the resulting embryo gestated by a female. He would then seek to assume parental rights over the child upon its birth. In this scenario, if rearing is considered to be within the definitional frame of reproduction, then the male’s “intent to parent” and the steps he takes towards bringing this situation about would count as “reproductive” actions. However, if rearing sits outside of the definitional frame of reproduction, then these actions would not count as reproduction and would therefore be ineligible to be afforded the sui generis and substantive liberty protections of his right to reproduce. If the reader accepts the reduced definitional scope of the term “reproduction” proposed in this paper, then it follows that the male’s rearing intent is not, in fact, covered by reproductive rights. I have argued that this is consistent with other forms of family formation, where the biological process of reproduction does not precede the rearing role, such as adoption or foster care. I suggest that including the intent and act of rearing a child as a part of reproductive liberty is conceptually confused. In other words, to include rearing within the scope of reproductive liberty is to overextend the conceptual framing of reproductive liberty.

There may indeed be other grounds upon which an individual could argue for a right to access ART, such as the right to found a family, but it is not upon reproductive grounds. This important point has been neglected in the literature to date because, I argue, the focus in this realm has remained on the rights of the hopeful parent—those involved in the non-sexual reproductive process who have an intention to rear the resulting child. However, an intention to rear does not generate a reproductive right to ART access. They can only ground such a claim in the arguably weaker moral claim of desiring to rear a child. Access to ART cannot be grounded in the assertion of reproductive rights in all instances. This paper will not critique the merits or moral standing of a “right to found a family” or a “right to rear a child” but only seeks to establish these as different rights to the reproductive right.

Robertson himself acknowledges, and then oddly dismisses, the fundamental theoretical distinction between reproducing and parenting: “Although childrearing is not, strictly speaking, reproduction, it is such an essential part of the reproductive experience that freedom to enter or leave the rearing role should be considered part of the freedom to procreate” (Robertson 1983, 410). I take issue with this view and argue that assigning *rearing* rights is a separate issue to determining who holds *reproductive* rights and obligations.

## Lack of intention to rear does not extinguish reproductive responsibility.

The second ethical implication of a stricter definition of reproduction is that all those who collaborate in the begetting and/or bearing stages of reproduction share the rights and responsibilities of reproduction, regardless of their intent—or lack of intent—to rear the resulting child. This is also regardless of whether their collaboration consists of genetic, gestational, or medical input. It is not controversial to suggest that reproduction generates, for those who reproduce, both rights and responsibilities to the child who is born. Indeed, this point is well established in the reproductive rights literature (Almond 2008; Benatar 2010). For example, O’Neill writes

… the right to beget or bear is not unrestricted, but contingent upon begetters and bearers having or making some feasible plan for their child to be adequately reared by themselves or by willing others. People who beget or bear without making any such plans cannot claim that they are exercising a right (O'Neill 1979, 25).

Others, such as Steinbock and McClamrock, develop a principle of parental responsibility where it could be morally wrong to intentionally conceive “when conditions are sufficiently awful that having children might be viewed as incompatible with being a good parent and unfair to the child” (Steinbock and McClamrock 1994, 15). This position holds that those involved in creating children have a degree of moral responsibility to consider the resulting child (Benatar 2010).

I accept this position in the literature, acknowledging that in the sexual realm this is limited to the female and male of the sexual union. However, in this paper I argue that the number of individuals who collaborate in the reproductive process expands in the move from sexual to non-sexual reproduction. In this realm, the number of people collaborating in the reproductive process at the point of begetting a child (triggering conception) expands to include fertility clinicians who provide ART.

I argue that having a “lack of intention to rear” the resulting child does not extinguish reproductive rights or responsibility. This is intuitively understood in the sexual realm, where the male and female of the sexual union are the sole collaborators in this reproductive process,^5^ and are the only two individuals to whom reproductive rights and responsibilities apply. Their intention or lack of intention to rear does not alter the normative assignment of reproductive rights and responsibilities upon them. Nor does their intent or lack of intent to rear alter the automatic conferral of parental recognition and responsibilities upon them when the child is born. Again, while rearing rights and responsibilities may flow on automatically from reproduction, this does not mean that they are the same.

In the non-sexual realm, collaboration in the stages of begetting and bearing expands. Begetting a child non-sexually requires fertility clinicians to join the process at this point of conception through the provision of ART treatment. The hopeful parent/s will also potentially require the collaboration of gamete donor/s and a surrogate to gestate and give birth to a child. These begetting and bearing roles can be completed by different individuals, some of whom have no intention to rear. I have argued in a previous paper that the rights and protections accorded to hopeful parents seeking to reproduce sexually are not sui generis reproductive rights at all (Hall 2022). Rather, reproduction is the assertion of three pre-existing and foundational moral rights: sexual freedom (to have sex, and to gestate for the female), bodily sovereignty (to have sex, to continue a viable pregnancy for the female), and the personal liberty to ask somebody to have sex with them (with reproductive intent). Indeed, I suggest that the reproductive right is no more than a right *to try to* reproduce, which is a necessarily weaker and narrower freedom to try to achieve anything. My point is that while a hopeful parent may seek to reproduce, unless they have the involvement of others (who are equally free to join the process, or not), they will not achieve this goal. If the person who is asked to have reproductive sex with a hopeful parent refuses to do so, there is—I have suggested—no violation of the hopeful parent’s reproductive right. It could be the assertion of their own (sexual) freedom to not have intercourse with the hopeful parent or of their bodily sovereignty to not engage in any of these proposed activities.

This rationale extends into the non-sexual reproductive realm and applies to all who join the process (Hall 2023). In other words, rights and responsibilities towards the child who is born are generated not only for the intended parents, but also for the gamete donors, surrogates, and clinicians who all collaborate in the creation of a child (even though none of these seek to rear the child they help create).^6^ Creating a child is an activity with a morally significant outcome, and on this basis, everybody who joins has the right to refuse to do so. All of these collaborators who beget the child have moral freedom to join the process, when asked, or to refuse. Joining is not a morally neutral act. The grounds upon which clinicians would be ethically justified in refusing differ from those who provide genetic or gestational input and would need to comply with existing professional and legal standards of anti-discrimination and equity. However, the basis upon which such refusal is ethically justified remains the same as for any reproductive collaborator. I additionally suggest that the state collaborates in non-sexual reproduction on the grounds that it regulates and in many jurisdictions subsidizes ART treatment. The state additionally has broadly recognized duties to consider future generations, which could include the future child born of ART treatment. However, the scope of this paper does not extend to substantive consideration of state-as-reproductive-collaborator, so I shall set this aside, while recognizing that such responsibilities to consider the welfare of future children may be relevant in relation to the state. Lack of an intention to rear the child has, I argue, shrouded the collaborative role of donor, surrogate, and clinician in the process to date in the literature, and the moral rights and responsibilities generated by these collaborative roles in ART have been ignored on the grounds that they do not seek to rear. I will briefly discuss the collaborative role of the fertility clinician, as the purpose of this paper is to explore the ethical implications of a revised definition of reproduction for ART provision.

### Clinician Collaboration in Non-Sexual Reproduction

The clinician has been framed as a morally neutral machine operator, of only instrumental significance in the reproductive process of hopeful parents. This is an understandable misconception, as the contribution of fertility clinicians does not create a biological connection with the future child. Nor do they intend to bear or rear the child they help to create. I recognize that clinician involvement in non-sexual reproduction not only differs from their role in the sexual realm but also differs from the role of hopeful parents (who intend to rear and beget and bear the child) and gamete donors or surrogates (who will beget and/or bear the child, thereby forming a biological connection with the child). I suggest that fertility clinicians are not “reproducers” in the same way as these other contributors are to the reproductive process. Their actions are not morally protected by any reproductive liberty assertion. However, I suggest that they nonetheless join the reproductive process through their involvement in performing ART interventions at the begetting stage of triggering conception.

I suggest that they “produce” a child through their collaboration. Their actions qualify for collaborative involvement, in line with the definition of causation as set out by Mackie (Mackie 1965). Applied to the fertility clinician’s role in the birth of a child via ART, I suggest that the involvement of a fertility clinician is an *unnecessary* condition for the conception of a child, but it is *sufficient* to contribute to the conception of a child non-sexually. Viewed from a different angle, the provision of ART by the fertility clinician is an *insufficient* but *necessary* part of the conception of a child non-sexually. On these grounds, I suggest that, similar to Mackie, the fertility clinician is likewise all of these things in triggering conception in the non-sexual reproductive process. These facts render the clinician a collaborator in the birth of children conceived non-sexually. I have argued in an earlier paper that, based on this collaborative involvement in a process with a morally important outcome, clinicians can ethically refuse to provide ART on three grounds: a) a conscientious objection asserted due to their personal code of ethics as an autonomous moral agent, b) professional duties of beneficence and non-maleficence to all who are implicated in or impacted by the treatment provided, and c) as agents of the state, carrying out their professional activities lawfully (Hall 2023).

The moral implications of a position that acknowledges a collaborative role of the clinician in triggering non-sexual reproduction could, and arguably should, have broader implications for the moral role of clinician involvement at other points in both sexual and non-sexual reproduction. For example, prior to the act of sexual intercourse which may lead to fertilization and implantation, clinicians are able to explore and sometimes treat physical problems affecting either or both sexual partners which may be preventing them from reproducing, such as treatment for malfunctioning pituitary glands or for polycystic ovaries, the prescription of ovarian hyper-stimulating drugs, or performing uterine ablation for females to remove cysts or scar tissue to prepare the uterus for implantation. Likewise, the points of gestation and childbirth can often benefit from substantial medical intervention which follows conception achieved via either means. While a substantive analysis of the moral role of reproductive collaborators at these other points on the reproductive continuum would be a valuable contribution to the literature of reproductive rights, I confine my analysis to an examination of the moral role of those who collaborate in triggering conception, as this is a fundamental difference between sexual and non-sexual reproduction.

Causal accounts of parenthood are extensively debated in the ART context in the literature (Nelson 2000; Bayne and Kolers 2003; Munson 1988; Callahan 1992; Fuscaldo 2006). These accounts largely seek to determine who, of those who create a child, has a greater parental claim over children they are involved in creating. Are the genetic ties morally binding, or more morally binding than gestational and/or intentional accounts of parenthood? However, prior to determining parental recognition for a child born via ART, I argue it is a more pressing moral task to take a step back and consider who, of all those who collaborate in the process, has moral responsibility for the creation of a child via ART. I suggest that regardless of whether or not they seek to stake a parental claim over the child, all those who collaborate in triggering conception of a child have moral rights and responsibilities towards that child. My point is that while it is important to establish parentage for every child born, it is equally important to establish who bears moral accountability for the child’s birth—that is, who has duties and obligations towards the future child. I argue these responsibilities are generated for anybody who collaborates in triggering the conception of a child. This is particularly so in the case of ART, where the creation of a child is both the intended and foreseeable outcome of the process, and includes the collaboration of clinicians through the provision of treatment. I suggest this has not been considered in the literature to date because the clinician has no intention to rear the child that is born. The literature focuses on the reproductive rights and duties of those with an intention to rear. I argue that it is morally unimportant to the reproductive process itself who intends to rear or who does not intend to rear the child that is created. If one’s actions or input are necessary conditions for triggering conception in either realm, I suggest that one has collaborated in the creation of any child that results from the conception.

## Conclusion

The term “reproduction” is limited to begetting and bearing children. While this mild definitional tweak has little impact in the realm of sexual reproduction, it has a significant impact on the formulation and scope of reproductive rights for hopeful parents, and perhaps even more substantive ethical implications for clinicians in the realm of non-sexual reproduction in two important ways. First, if one does not beget or bear a child, one does not reproduce. Second, those who collaborate in the reproductive process bear reproductive rights and duties, even if they do not seek to rear the child they help create.

The purpose of this paper is to highlight a significant theoretical inconsistency in the literature of reproductive rights: rearing does not form part of reproduction. The two substantial ethical implications of this have been shown to be twofold: 1. Access to ART cannot be grounded in a hopeful parent’s reproductive right if they do not beget or bear the child. 2. Reproductive rights are not extinguished by lack of intention to rear. Further to this point, I have demonstrated that clinicians collaborate in non-sexual reproduction through the provision of ART and are therefore morally permitted to agree or refuse to be involved, as are all reproductive collaborators.

## Footnotes

1 In this paper I do not explore the relationship between begetting and bearing a child with the assignment of rearing rights or obligations.

2 1965. Griswold v. Connecticut. U.S. 1972a. Eisenstadt v. Baird. U.S. 1973. Roe v. Wade. U.S.

3 1923. Meyer v. Nebraska In 262: U.S. 1925. Pierce v. Society of Sisters U.S 1972b. Wisconsin v. Yoder. U.S. 2000. Troxel v. Granville. U.S.

4 This paper is a theoretical analysis of the implications of removing “rearing” from the definition of “reproduction.” It is not an analysis of the potential inequities faced by people trying to build families via ART in relation to the cost of ART, or who pays for it. However, I recognize this would be an important contribution to the literature.

5 I recognize that there can be high levels of medical assistance at the pre-conception point by gynaecologists and other fertility experts and during gestation and childbirth with the assistance of obstetricians, midwives, and doulas, including surgical interventions in utero. While it could equally be argued that these assistors provide collaborative input into the reproductive project at these stages, this paper is concerned with those who collaborate in begetting a child at the point of triggering conception.

6 I suggest that gamete and embryo donors and surrogates bear a measure of moral responsibility for the child born of their donation, mirroring the responsibilities generated for the genetic and gestational collaborators who seek to bear the child’s parents following childbirth, and on the same grounds that all have a physical connection with the child who is created. Due to space constraints, I will not discuss their involvement or moral responsibility towards the child who is born any further.
